# Targeted control of pneumolysin production by a mobile genetic element in *Streptococcus pneumoniae*

**DOI:** 10.1099/mgen.0.000784

**Published:** 2022-04-13

**Authors:** Emily J. Stevens, Daniel J. Morse, Dora Bonini, Seána Duggan, Tarcisio Brignoli, Mario Recker, John A. Lees, Nicholas J. Croucher, Stephen Bentley, Daniel J. Wilson, Sarah G. Earle, Robert Dixon, Angela Nobbs, Howard Jenkinson, Tim van Opijnen, Derek Thibault, Oliver J. Wilkinson, Mark S. Dillingham, Simon Carlile, Rachel M. McLoughlin, Ruth C. Massey

**Affiliations:** ^1^​ School of Cellular and Molecular Medicine, University of Bristol, Bristol, BS8 1TD, UK; ^2^​ Centre for Ecology and Conservation, University of Exeter, Penryn Campus, Exeter, TR10 9FE, UK; ^3^​ Institute of Tropical Medicine, University of Tübingen, Tübingen, Germany; ^4^​ MRC Centre for Global Infectious Disease Analysis, Department of Infectious Disease Epidemiology, St. Mary’s Campus, Imperial College London, London, W2 1PG, UK; ^5^​ The Wellcome Trust Sanger Institute, Wellcome Trust Genome Campus, Hinxton, Cambridge, CB10 1SA, UK; ^6^​ Big Data Institute, Nuffield Department of Population Health, University of Oxford, Oxford, OX3 7LF, UK; ^7^​ Bristol Dental School, University of Bristol, Bristol, BS1 2LY, UK; ^8^​ Biology Department, Boston College, Chestnut Hill, MA, USA; ^9^​ DNA-Protein Interactions Unit, School of Biochemistry, University of Bristol, Bristol, BS8 1TD, UK; ^10^​ Host Pathogen Interactions Group, School of Biochemistry and Immunology, Trinity Biomedical Sciences Institute, Trinity College Dublin, Dublin 2, Ireland; ^11^​ Schools of Microbiology and Medicine and APC Microbiome Ireland, University College Cork, Cork, Ireland

**Keywords:** *Streptococcus pneumoniae*, ICE elements, pneumolysin regulation, ZomB protein

## Abstract

*
Streptococcus pneumoniae* is a major human pathogen that can cause severe invasive diseases such as pneumonia, septicaemia and meningitis. Young children are at a particularly high risk, with an estimated 3–4 million cases of severe disease and between 300 000 and 500 000 deaths attributable to pneumococcal disease each year. The haemolytic toxin pneumolysin (Ply) is a primary virulence factor for this bacterium, yet despite its key role in pathogenesis, immune evasion and transmission, the regulation of Ply production is not well defined. Using a genome-wide association approach, we identified a large number of potential affectors of Ply activity, including a gene acquired horizontally on the antibiotic resistance-conferring Integrative and Conjugative Element (ICE) ICE*Sp*23FST81. This gene encodes a novel modular protein, ZomB, which has an N-terminal UvrD-like helicase domain followed by two Cas4-like domains with potent ATP-dependent nuclease activity. We found the regulatory effect of ZomB to be specific for the *ply* operon, potentially mediated by its high affinity for the BOX repeats encoded therein. Using a murine model of pneumococcal colonization, we further demonstrate that a ZomB mutant strain colonizes both the upper respiratory tract and lungs at higher levels when compared to the wild-type strain. While the antibiotic resistance-conferring aspects of ICE*Sp*23FST81 are often credited with contributing to the success of the *S. pneumoniae* lineages that acquire it, its ability to control the expression of a major virulence factor implicated in bacterial transmission is also likely to have played an important role.

## Data Summary

**Impact Statement**

Bacterial pathogens regulate the expression of their virulence factor to maximise their long-term survival and transmission. In this study we sought to identify novel regulators of the expression of pneumolysin (Ply), a cytolytic toxin secreted by the major human pathogen *Streptococcus pneumoniae *. Using a GWAS approach we identified a number of loci associated with Ply production, including a gene we have named *zomB* that is located on an Integrative and Conjugative element (ICE*Sp*23FST81). This mobile genetic element was believed to enhance the success of the pneumococcal lineage that acquired it, due to the antibiotic resistance genes it brought with it. However, our findings suggest that the presence of *zomB* on this ICE element also increased the transmissibility of this lineage by causing a signficant increase in the expression of Ply.

Supplementary data is provided in the online version of this article, including two supplementary figures and four spreadsheet containing details of Ply activity, antibiotic resistance and accession numbers for the genome sequence of the clinical strains used here; and the GWAS outputs from the three methods used. The accession number for the RNAseq data is provided within the main text of the article.

## Introduction

For opportunistic pathogens, such as *Streptococcus pneumoniae *, there is a fine balance to be reached between the ability to colonize the host asymptomatically, to transmit between hosts, and to cause disease [1–3]. The secretion of cytolytic toxins is often key to this. *S. pneumoniae,* for example, produces pneumolysin (Ply), a cytolytic pore-forming toxin that binds to cholesterol in the membranes of host cells, where it inserts into the lipid bilayer forming a transmembrane pore that lyses the host cell [4–7]. Ply also affects the host immune system in a complex manner, with evidence for both pro- and anti-inflammatory activity [8–11]. The data surrounding the role of Ply in nasal colonization are complex, with early studies suggesting it contributes positively to the colonization process [12], but more recent work has shown its expression to be inversely correlated with colonization duration and directly correlated with shedding of *S. pneumoniae* from the nose for transmission to new hosts [13–15]. Given the importance of Ply to many aspects of the biology of *S. pneumoniae,* it represents an attractive target for the development of therapeutic intervention [16].

Despite its importance, what is perhaps surprising about Ply is that so little is known about the regulation of its production compared to the virulence factors of similar pathogens, such as *Staphylococcus aureus* [17]. The *ply* gene is transcribed as part of an operon with four other genes [18, 19]. One gene encodes a transcriptional regulator *yebC* [20] (although this is labelled as *yeeN* on Pneumobrowser [21]), and its effect on the transcription of the *ply* operon is not known. The activity of the proteins encoded by the other three genes are also not understood, although they have been implicated in the movement of Ply from the cytosol to the bacterial cell wall [18, 19]. Microarray-based transcriptional analyses suggest that *ply* is transcribed in a density-dependent manner, where its transcription was significantly lower in a LuxS quorum sensing mutant [22]. This supports earlier work based on the haemolytic activity of the bacteria that reported maximal Ply activity in the late exponential phase of growth [23, 24]. Interestingly, *ply* has also been shown to be expressed during the early phases of biofilm development in a LuxS-dependent manner, although the role it plays under these growth conditions has yet to be defined [25]. Other microarray work also suggests that RR09, the response regulator of the TC09 two-component regulatory system, down-regulates *ply* transcription under specific growth conditions [26]. Once transcribed and translated, Ply accumulates in the cytoplasm as it does not contain a secretory signal sequence, and its release has been shown to be dependent upon the major autolysin for *S. pneumoniae,* LytA [27]. There have, however, also been reports of an autolysis-independent, cell-contact-mediated release of Ply from the bacterial cells, although little is known about the mechanistic detail underpinning this aside from the involvement of an accessory secretory system [22, 28].

Given how poorly understood the regulation of the production of this major virulence factor is, here we sought to define the genetic basis of Ply activity by analysing a collection of 165 isolates belonging to the *S. pneumoniae* Pneumococcal Molecular Epidemiology Network 1 (PMEN1) lineage [29]. This globally successful clonal group (also known as vaccine serotype 23F, multilocus sequence type 81) is of public health importance having made an important contribution to the emergence of penicillin non-susceptibility amongst the pneumococci [30]. It has acquired additional antibiotic resistance through the acquisition of the Integrative and Conjugative Element (ICE), ICE*Sp*23FST81 [8], which confers resistance to tetracycline and chloramphenicol, features believed to have contributed to the success of this lineage. As such it represents an important and relevant lineage on which to base this study.

## Methods

### Bacterial strains and growth conditions

The clinical isolates used in this study (listed in Table S1, available in the online version of this article) had been previously described and sequenced [29] and all belonged to the PMEN1 clone of which *S. pneumoniae* ATCC 700669 is the reference strain. This collection contains global strains that were isolated from a range of pneumococcal diseases between 1984 and 2008 [Table S2 in reference 29]. Strains were grown for 16–24 h in 5 % CO_2_ at 37 °C, either on brain-heart infusion (BHI), on Todd Hewitt supplemented with 0.5 % (w/v) yeast extract (THY), on blood agar plates containing 5 % (v/v) defibrinated horse blood or in BHI/THY broth without blood.

### Construction of deletion mutants in *S. pneumoniae*

Genes were deleted in *S. pneumoniae* using linear PCR products as described previously [31]. In brief, a stitch PCR approach using the primers listed in Table 1 were used to generate a single PCR product consisting of 1 kb of DNA to either side of the gene to be deleted, with a gene encoding resistance to erythromycin (*ermAM*; amplified from plasmid pVA838) in the centre. To transform the bacteria with this PCR product, the wild-type bacteria were grown overnight in 5 ml BHI broth, and 2 ml of this culture was used to inoculate a pre-warmed tube of 20 ml BHI broth. This was incubated for a further 2 h, and 0.5 ml of the culture was added to 9.5 ml pre-warmed BHI broth and incubated for 30 min. Then, 1 ml of this sub-culture was transferred to sterile tubes, 10 µl of 10 µg ml^−1^ competence-stimulating peptide-2 (CSP-2) was added to each tube, and these were incubated for 15 min. To 200 µl aliquots of this, either the PCR product or sterile water was added, and the mixture incubated for a further 2 h. This was then added to molten BHI agar (10 ml) containing 5 % (v/v) defibrinated horse blood, allowed to set in a Petri dish and incubated for 2 h. Plates were then overlaid with a further 10 ml molten agar containing 5 % (v/v) defibrinated horse blood with erythromycin (2 µg ml^−1^) to selected for successfully transformed cells. Plates were incubated for up to 5 days at 37 °C, or until colonies appeared within the agar.

**Table 1.** Primers used to construct mutants

Amplification site	Primer sequence
*ply* (LHS) forward	5′ CCCTTGCTCTGGTTAAAAAAAGAAGC 3′
*ply* (LHS) reverse	5′ ATATTTTTGTTCATATTTGCCATCTTCTACC 3′
*ery-ply* forward	5′ GGTAGAAGATGGCAAATATGAACAAAAATATAAAA 3′
*ery-ply* reverse	5′ CTACCTGAGGTTATTTCCTCCCGTT 3′
*ply* (RHS) forward	5′ GAGGAAATAACCTCAGGTAGAAGATAAG 3′
*ply* (RHS) reverse	5′ GATCACCTTTTTTAGCTGCTACATAG 3′
*zomB* (LHS) forward	5′ TGCCCACTATTTTTATCTAGTTGCTTACC 3′
*zomB* (LHS) reverse	5′ ATTTTTGTTCATTGTTGTCATCGTTTTACCTC 3′
*ery-zomB* forward	5′ CGATGACAACAATGAACAAAAATATAA 3′
*ery-zomB* reverse	5′ CTTTTCCGGATTCTTATTTCCTCCC 3′
*zomB* (RHS) forward	5′ GGAGGAAATAAGAATCCGGAAAAG 3′
*zomB* (RHS) reverse	5′ AATTAATTCCTGAATACAAGTTAACAAAATAG 3′

### Cloning of ZomB for complementation

The *zomB* gene was amplified by PCR from strain ATCC 700669 using KAPA HiFi HotStart ReadyMix (Roche) and primers zomBFW: ATATGCATGCCCTCGTAATTACTAGGAAAC (*Sp*HI, *T*_m_ 67.6 °C) and zomBRV: ATATGGATCCATTTTCTTATCTTATAGATTCTAAAATAC (*Bam*HI, *T*_m_ 63.7°C) and cloned into the pVA838 plasmid [32] using MAX Efficiency DH5α Competent Cells (Invitrogen) to make pVA838-zomB. The plasmid was purified and transformed into D39 as described above, using CSP-1 in place of CSP-2.

### Quantification of Ply activity

Strains were grown overnight in BHI broth, then sub-cultured at a 1 : 1 ratio into fresh broth and grown for 1 h until an OD_600nm_ of 0.4–0.7 was reached. These cultures were then serially diluted 4-fold in a 96-well plate containing BSA assay buffer (50 mg BSA and 77 mg DTT dissolved in 50 ml sterile PBS). To each well, 50 µl of triple-washed sheep red blood cells (at a final concentration of 2 % diluted in PBS, v/v) was added and the plates were incubated for 1 h in 5 % CO_2_ at 37 °C. The plates were then centrifuged at 2000 r.p.m for 10 min at room temperature to separate the intact cells from the soluble lysed material. The supernatants from each well were transferred to a fresh 96-well plate and absorbance values read at 415 nm were obtained using a FLUOstar Omega microplate reader.

### Genome-wide association study (GWAS)

The initial genome-wide associations between SNPs and bacterial toxicity were determined by means of linear regression. To account for bacterial population structure, we first performed a singular value decomposition [principal components analysis (PCA)] of the SNP data and then used the first four principal components (PC), which together explained around 45 % of the variance, in the regression model:

.$$toxicity\;∼\;\beta_{0}+\beta_{1}SNP\;+\;\beta_{2}PC_{1}+\beta_{3}PC_{2}+\beta_{4}PC_{3}+\beta_{5}PC_{4}$$

Statistical significance of the $\beta_{1}$ term is reported at an uncorrected α=0.05 threshold. The risk of generating false positive results by not correcting for multiple testing was deemed preferable over the increased likelihood of false negative results, which could potentially discard important and novel associations, particularly given all findings were subsequently validated by mutating the target site. Additional GWAS analyses were performed using pyseer and BugWas as previously described [33, 34].

### Quantification of Ply production

The bacteria were grown overnight in 15 ml BHI broth and harvested by centrifugation. Six millilitres of bacterial culture supernatant was concentrated to 500 µl using a Pierce Protein Concentrator PES, 10k molecular weight cutoff (Thermo Fisher Scientific). The individual proteins were separated on SDS-PAGE gels and Western blotting of this was conducted using anti-pneumolysin antibody followed by goat anti-mouse IgG horseradish peroxidase (HRP) as the secondary antibody (both from Abcam). The HRP signal was detected using the Metal Enhanced DAB Substrate Kit (Thermo Fisher Scientific).

### Quantification of transcription of the *ply* gene

Bacteria were grown overnight in 5–10 ml BHI broth and RNA was extracted using a Zymo Quick-RNA Fungal/Bacterial Miniprep kit following the manufacturer’s protocol. A Turbo DNase digest kit was used to remove any contaminating DNA. The quality and quantity of the RNA were determined using a Nanodrop and the RNA was reverse transcribed to cDNA using a qScript cDNA synthesis kit (QuantaBio), as per the manufacturer’s protocol. RNA samples were standardized such that 100 ng was added to each cDNA synthesis reaction. Quantitative reverse transcriptase PCR (qRT-PCR) was performed using a Mic qPCR cycler (Bio Molecular Systems) and reactions were set up using a KAPA Sybr Fast universal master mix (no rox). Three technical repeats were conducted for each cDNA sample and the data were analysed using the 2^−(ΔCt ply – ΔCt recA)^ method [35]. The cycle parameters included an initial denaturation at 95 °C for 3 min, followed by 35 cycles of 95 °C for 10 s, annealing at 60 °C for 20 s and elongation at 72 °C for 20 s. A melt curve analysis was performed to check the amplified products. The primer pairs where *recA* was used as the control housekeeping gene were: ply FW, GGCACCACTATGATCCAGCA; ply RV, CAGGCAAGGTGGACATGGTA; recA FW, ATCGGAGATAGCCATGTTGG; and recA RV, ATAGAGGCGCCAAGTTTACG.

### Expression and purification of ZomB

The *zomB* gene was cloned into the expression plasmid pET15b, and expressed in *Escherichia coli* strain BL21(DE3) with a 6× histidine tag at its N terminus. The *E. coli* cells were grown at 37 °C in LB broth containing 100 µg ml^−1^ ampicillin to an OD_600_ of ∼0.5, whereupon cells were temperature acclimatized to 18 °C and gene expression was induced for 12 h via the addition of 1 mM IPTG. Cells were harvested by centrifugation for 20 min at 6000 r.p.m. and resuspended in buffer containing 50 mM Tris pH 7.5, 150 mM NaCl, 10 % sucrose and 0.1 mM PMSF. Cells were lysed by sonication in buffer containing 1 mM Tris(2-carboxyethyl) phosphine (TCEP), 500 mM NaCl, 20 mM imidazole and a protease inhibitor cocktail, and cell debris was removed by centrifugation for 30 min at 21 500 r.p.m. Cell lysate was loaded onto a 5 ml HisTrap Nickel binding column which had been equilibrated in HisB buffer (20 mM Tris pH 7.5, 500 mM NaCl, 1 mM TCEP, 5 % glycerol) plus 20 mM imidazole. Proteins possessing His-tags were eluted with a gradient from 20 to 500 mM imidazole over 32 min with a flow rate of 3 ml min^−1^. Protein elution was monitored by measuring absorbance at 280 nm. Fractions containing the most protein were pooled and applied to a HiTrap Heparin affinity column equilibrated with HepQ/B buffer (20 mM Tris pH 7.5, 1 mM TCEP) plus 100 mM NaCl. Heparin-binding proteins were eluted with a gradient from 100 mM to 1 M NaCl over 30 min, monitored by absorbance at 280 nm. Two millilitre fractions containing the highest protein concentrations were pooled and applied to a MonoQ anion exchange chromatography column equilibrated with HepQ/B buffer plus 100 mM NaCl. Proteins were eluted with a gradient from 100 mM to 1 M NaCl over 30 min, with protein absorbance monitored at 280 nm, and 0.3 ml fractions were collected. Fractions containing the highest concentrations of eluted protein were pooled and stored in HepQ/B buffer plus 330 nM NaCl, corresponding to the salt concentration at which the protein was eluted from the column. Nanodrop OD_280_ and the ZomB protein’s predicted extinction coefficient (118390 mol l^–1^ cm^–1^) were used to determine that ZomB had been purified to a concentration of 7.0 µM.

### Biochemical characterization of ZomB activity

ATPase activity was measured by coupling the hydrolysis of ATP to the oxidation of NADH, which gives a change in absorbance at 340 nm. Reactions were performed in a buffer containing 20 mM Tris-Cl pH 8.0, 50 mM NaCl, 2 mM DTT, 1 mM MgCl_2_, 50 U ml^−1^ lactate dehydrogenase, 50 U ml^−1^ pyruvate dehydrogenase, 1 mM phosphoenolpyruvate and 100 µg ml^−1^ NADH. Rates of ATP hydrolysis were measured over 1 min at 25 °C and the ssDNA substrate used was Poly(dT). For calculation of *K*_DNA_ (defined as the concentration of DNA at which ATP hydrolysis is half-maximal), the ATP concentration was fixed at 2 mM. The Michaelis–Menten plot was performed at a saturating DNA concentration, which is defined as 10 times the *K*_DNA_ value. The concentration of ZomB was 50 nM in these assays unless indicated otherwise.

### Electrophoretic mobility shift assays

The intergenic region between *ply* and the neighbouring gene SPN23F19460, containing BOX repeat regions, was amplified (using the following primers: BoxF, GAGAGGAGAATGCTTGCGAC; and BoxR, TAGGAATCTCCTTTTTTCACATTTTAATCTTTC). A region of the *ply* gene of equivalent size was also amplified (using PlyF, ATGGCAAATAAAGCAGTAAATGACTTTATAC; and

PlyR, GCCCCCTAAAATAACCGCCTTC). The purified ZomB protein was added in a range of concentrations (5, 2.5, 1.25, 0.625, 0.3125 and 0.15625 µM) to 10 nM of the PCR products in a buffer containing 20 mM Tris (pH 8), 200 mM sodium chloride, 1 mM TCEP and 10 % glycerol. Samples were then run on a 1.5 % agarose gel in 1× TAE buffer for 110 min at 90 V. Following this, the gel was stained in TAE containing 1× SYBR Safe DNA gel stain for 30 min, and bands were visualized using a Typhoon FLA 9500.

### RNA sequencing

Three independent 20 ml cultures of *S. pneumoniae* ATCC 700669 wild-type and *ΔzomB* mutant were grown overnight in THY broth, from which total RNA was extracted and DNase treated as described above. RNA was stored at −70 °C until transportation on ice for sequencing at the University of Bristol Genomics Facility. RNA integrity was determined by electrophoresis using a TapeStation (Agilent) RNA Screentape Assay, and samples with scores of >7 were considered suitable for library preparation and sequencing.

One hundred nanograms of total RNA was taken into the Illumina TruSeq Stranded Total RNA with Illumina Ribo-Zero Plus rRNA Depletion kits according to the manufacturer’s instructions. Briefly, the protocol involved enzymatic depletion of rRNA and clean-up of the remaining RNA using magnetic beads. The RNA was fragmented and denatured, and first- and second-strand cDNA was synthesized, then total cDNA was purified using magnetic beads. The 3′ ends were adenylated to prevent blunt end ligation, and indexing adapters were ligated to the ends of the double-stranded cDNA fragments. Magnetic beads (Agencourt AMPure XP beads; Beckman Coulter) were then used to clean up the cDNA libraries before amplification of DNA fragments, selecting for adapter molecules. A second clean up with magnetic beads was performed, and the libraries were quantified using the ThermoFisher High Sensitivity dsDNA Qubit assay and validated using the TapeStation (Agilent) with the DNA100 screentape assay. The cDNA libraries were normalized to 4 nM and pooled for sequencing on the Illumina NextSeq500 instrument using a High Output Version 2.5 sequencing kit. The indices for each sample are detailed in Table 2. The depth of sequencing covered 2×75 bp paired-end reads, with a minimum of 30 million reads per sample. Sequence information was output in FastQ file format for subsequent downstream analysis.

**Table 2.** Sample indices for sequencing

Sample	Index 1 (i7)	Index 2 (i5)
WT1	CCGCGGTT	CTAGCGCT
WT2	TTATAACC	TCGATATC
WT3	GGACTTGG	CGTCTGCG
zomB1	AAGTCCAA	TACTCATA
zomB2	ATCCACTG	ACGCACCT
zomB3	GCTTGTCA	GTATGTTC

### Bioinformatic analysis of RNA-sequencing data

FASTQ output files from the Illumina sequencing platform [four files for each forward and reverse read (one for each lane used in sequencing)] were concatenated into a single file for each forward and reverse read for each sample. The FASTQ files were checked for quality using FASTQC (version 0.11.9; Brabraham Bioinformatics), ensuring consistency and high-quality scores, particularly within ‘per base sequence quality’ and ‘per sequence quality score’ tabs. These files were then taken through a bioinformatics pipeline (via a terminal on an Apple MacBook pro running macOS Catalina, version 10.15.7) as detailed below.

An index for the *S. pneumoniae* reference genome (GenBank accession GCA_000026665.1) was created using Bowtie 2 [35] (version 2.4.1), and FASTQ files were aligned to this also using Bowtie 2. The overall alignment rate for the samples was between 87.54 and 97.28 %. The annotated genome was then used to create a sequence alignment map (SAM) using SAMtools [36] (version 1.11). Here, a quality control step involved viewing the header of the files to check for quality scores and correct file format. The SAM files were sorted and converted to binary alignment map (BAM) files using SAMtools. The annotated genome was then used as a reference for counting the sorted BAM file reads using Subread featureCounts [37] (version 2.0.1) including options for paired-end reads.

The featureCounts output file was imported into RStudio (version 1.2.5033) for differential expression analysis using DESeq2 [38] (version 1.26.0). The RStudio source code is available upon request. The aligned BAM files and reference can be obtained at the NCBI Sequence Read Archive (SRA) under accession PRJNA706751.

### Mouse intranasal challenge

Eight- to 10-week-old female C57/Bl6 mice were inoculated intra-nasally with 1×10^6^ c.f.u. of WT, or an isogenic ZomB-deficient strain under isoflurane anaesthetic. Mice were killed on days 1, 3 and 7 post-inoculation. The upper respiratory tract was lavaged by the insertion of a 20-gauge intravenous catheter into the trachea with 1 ml of sterile PBS washed through and collected at the nose. Lungs were removed and homogenized in 1 ml of sterile PBS. Lung homogenate and nasal lavage were plated on BHI agar with 5 % (v/v) defibrinated horse blood and 2.5 µg ml^−1^ tetracycline for enumeration of c.f.u.

## Results

**Fig. 1.**
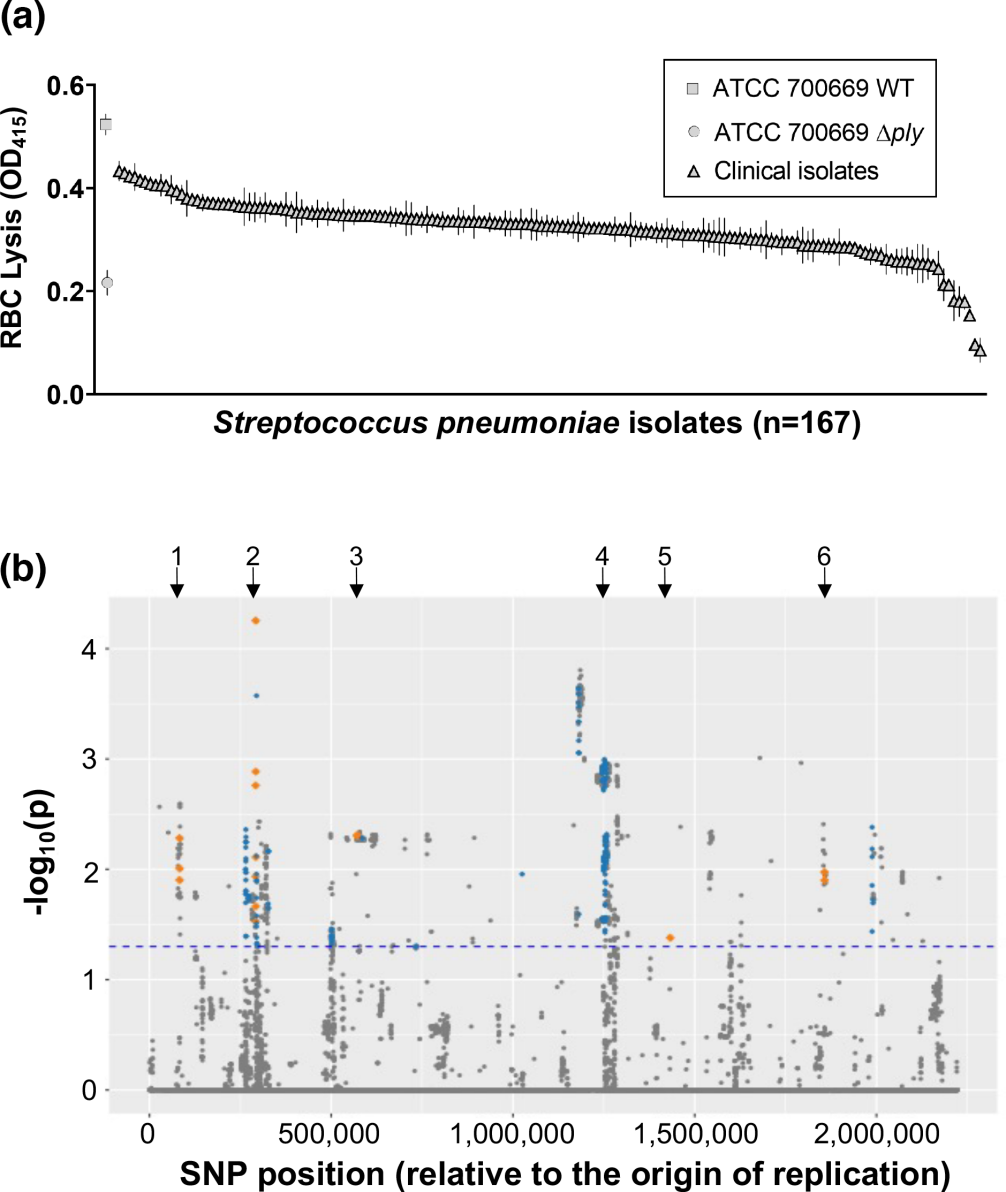
GWAS to identify novel effectors of Ply production by *S. pneumoniae *. (**a**) Pneumolysin activity of 165 *S *. *pneumoniae* of the PMEN1 lineage, as measured by cell lysis. A wild-type (WT) and isogenic pneumolysin mutant (Δ*ply*) were included as controls. (**b**) Manhattan plot of the SNP-based GWAS. The horizontal blue dotted line indicates the threshold for significance (not corrected for multiple tests). SNPs in loci identified by two or all three GWAS methods are indicated in blue and orange respectively. The black arrows indicate the regions of interest: 1, SPN23F00840; 2, *pbpX*; 3, intergenic region between SPN23F05820 and *bgaA*; 4, ICE*Sp23*FST81; 5: intergenic region between SPN23F14800 and *greA*; and 6, intergenic region between SPN23F19120 and *msmG*.

To examine what variation exists in the production of Ply across a collection of closely related *S. pneumoniae* clinical isolates, we first constructed a *ply* mutant in the pneumococcal PMEN1 isolate ATCC 700669 [8] by replacing the *ply* gene with an erythromycin resistance cassette. Using this wild-type and mutant strain pair as positive and negative controls, we quantified the Ply activity [lysis of sheep red blood cells (RBCs)] of 165 PMEN1 clinical isolates in triplicate, which revealed significant variation across this collection of closely related isolates (Fig. 1a). As the genomes of each of these isolates have been sequenced [29], we applied three complementary GWAS approaches to identify loci associated with Ply activity. In addition to a linear regression approach using the SNP data, we also applied two methods that make use of kmer (lengths of nucleotide sequences) data: *BugWAS* [39] and *pyseer* [33]. The results from each of these methods are presented in Tables S1–S3. A Manhattan plot in Fig. 1(b) shows the significantly associated genetic loci determined through the SNP-based method, highlighting those loci where two or all three GWAS methods agreed. (Note, the *P* values are not comparable between the three methods, so this graph is only provided as an illustration of the genomic location of the commonly associated loci.) There were two notable observations from these analyses. The first was that five loci were associated with Ply activity across all three methods, with the *pbpX* gene, which encodes the Penicillin Binding Protein 2x, being the most significant. This was followed in order of significance by the intergenic region between a gene with the locus tag SPN23F05820 and *bgaA,* a gene with the locus tag SPN23F00840, the intergenic region between a gene with the locus tag SPN23F19120 and *msmG,* and the intergenic region between a gene with the locus tag SPN23F14800 and *greA*. The second notable observation was that 48 individual genes or intergenic regions on ICE*Sp23*FST81 were associated with Ply activity.

Fitness trade-offs between antibiotic resistance and virulence in bacteria are well established [20, 40–42], and for both clinical isolates and isogenic mutants the acquisition of penicillin resistance has been shown to decrease the virulence of *S. pneumoniae* in murine models of infection [43, 44]. Given the association of the *pbpX* gene and Ply activity, we hypothesized that a similar trade-off may be occurring here, such that the polymorphisms in the *pbpX* gene may be increasing the levels of resistance to penicillin, which may consequently reduce the levels of Ply being produced, or vice versa. Although bacterial GWAS results are typically validated through mutation of the associated locus, the contribution of the protein encoded by *pbpX* to the biosynthesis of the peptidoglycan layers in the bacterial cell wall is such that it is essential and cannot be inactivated. Instead, we tested our hypothesis by examining the levels of resistance to penicillin (minimum inhibitory concentrations, MICs) for the isolates, but found no significant correlation between the MICs and Ply activity (Pearson product-moment correlation *r* ^2^=0.076, *P*=0.36).

**Fig. 2.**
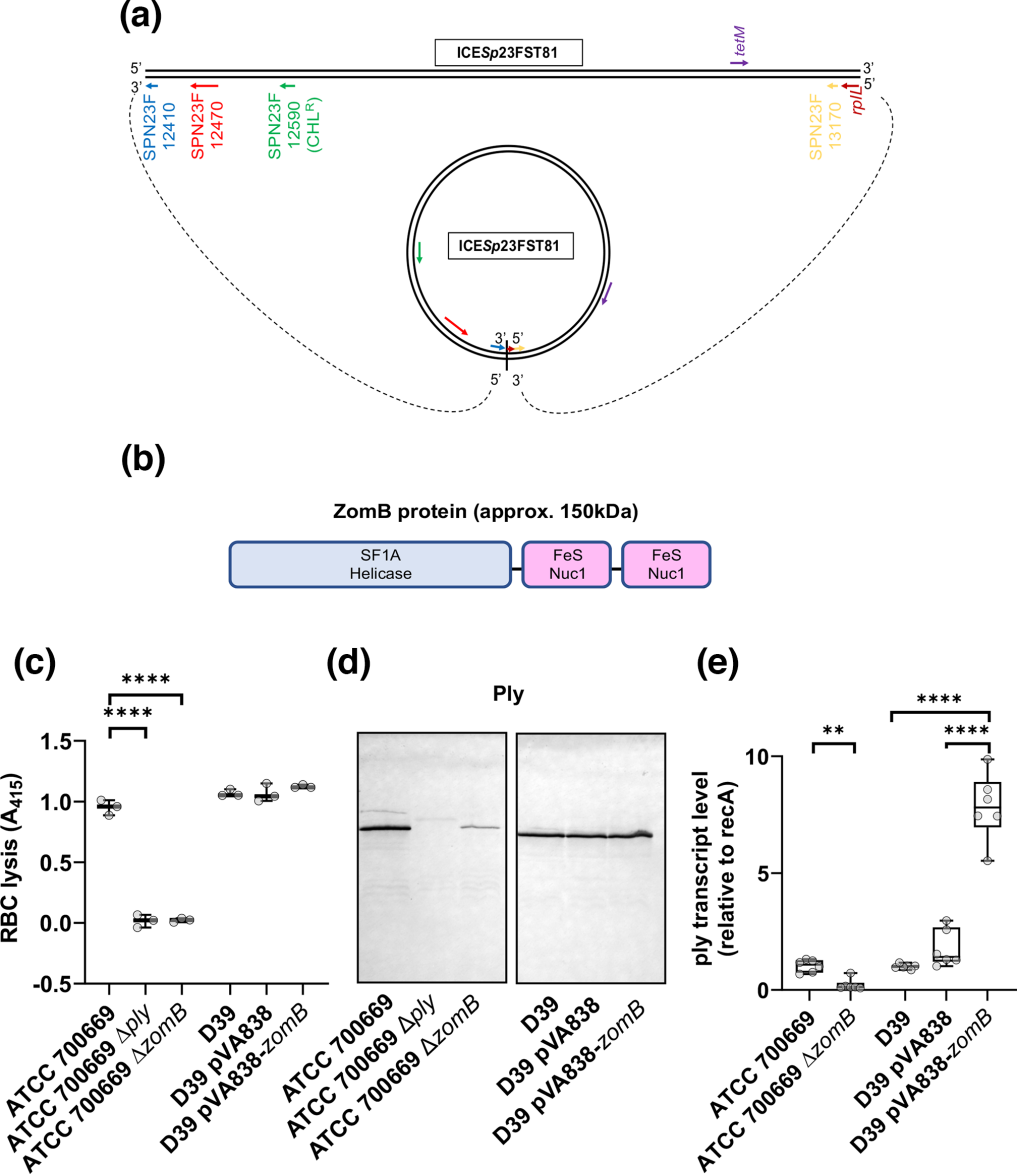
Inactivation of the *zomB* gene on ICE*Sp23*FST81 affects pneumolysin production. (**a**) Cartoon illustration of ICE*Sp23*FST81 in both its linear chromosomally integrated form and circularized plasmid form. Some genes of interest have been included: SPN23F12410, SPN23F13170 and *rplL* because these flank the element in its linear form and come into close association when in plasmid form. Genes coding for antibiotic resistance to chloramphenicol and tetracycline are also indicated. (**b**) Schematic of the ZomB protein with its helicase and two Cas4-like nuclease domains indicated. (**c**) The lytic activity of the *zomB* mutant was comparable to that of the *ply* mutant as determined by a sheep RBC lysis assay in strain ATCC 700669. In strain D39, introduction of the *zomB* gene on the pVA838 plasmid did not affect the RBC lytic activity of the bacteria. (**d**) The presence of Ply in the extracellular medium was reduced in the *zomB* mutant of strain ATCC 700669, detected using anti-Ply antibodies in a Western blot on concentrated bacterial supernatant. In strain D39, introduction of the *zomB* gene on the pVA838 plasmid did not affect the level of Ply production. The commassie stained gel verifying equal protein loading can be found in Fig. S2. (**e**) The deletion of *zomB* in strain ATCC 700669 reduced the transcription of the *ply* gene, and introduction of the *zomB* gene into strain D39 increased *ply* transcription. The transcription of *ply* was determined by qRT-PCR, where the data were made relative to the expression of the housekeeping gene *recA* in each sample and normalized to the level of expression on the *ply* gene in the wild-type strain. The box plots represent the median and interquartile ranges; individual data points are indicated by open circles.

The high number of associated loci on ICE*Sp*23FST81 is particularly intriguing. This mobile genetic element (MGE), which can be found both integrated and in plasmid form (illustrated in Fig. 2a), is believed to be critical to the success of this lineage of *S. pneumoniae* due to the antibiotic resistance capabilities it brings to the bacteria [8]. Given its ability to move horizontally as a single contiguous unit between bacteria, it is probable that of the associated loci only one is an affector of Ply activity, whilst the others are associated through their physical linkage to this. A survey of the putative activity of all 48 associated loci revealed that the majority of these are genes typical of such elements, involved in antibiotic resistance and the mechanics of its movement. An interesting exception is a gene with the locus tag SPN23F12470. This locus has been annotated as encoding a UvrD-like helicase, a family of proteins typically associated with core housekeeping activities for bacteria. Further *in silico* analysis of the encoded protein, which we have named ZomB, suggests that it is a multi-domain protein with a putative DNA binding helicase domain, followed by two Cas4-like nuclease domains which are predicted to harbour 4Fe-4S clusters [45] (Fig. 2b). With several known examples of Cas-like proteins regulating bacterial virulence [46–48], we hypothesized that it is this gene on ICE*Sp*23FST81 that is the affector of Ply activity. Further analysis of the sequence data revealed six isolates with non-synonymous SNPs in the *zomB* gene and all six were amongst the top 50 % of haemolytic isolates (Fig. S1). The SNPs conferred the following amino acid changes: T27M, A122T, D353N and L685F, all of which reside in the putative helicase region of the ZomB protein.

To establish the role of ZomB in Ply activity, we replaced the *zomB* gene with an erythromycin resistance cassette in *S. pneumoniae* strain ATCC 700669. While no differences in growth between the wild-type and mutant strains was observed, the RBC lytic activity of the *zomB* mutant was significantly impaired relative to the wild-type strain (Fig. 2c). Using anti-Ply antibodies in a Western blot to understand the mechanism by which ZomB affects Ply activity, we found that this reduction in lytic activity was due to a decrease in the amount of Ply protein being released into the bacterial culture supernatant (Fig. 2d). We also quantified the relative transcription of the *ply* gene by qRT-PCR and found that the reduced abundance of Ply was due to a significant decrease (19-fold) in *ply* transcription when the *zomB* gene was inactivated (Fig. 2e). Despite numerous attempts to complement this mutation, we were unable re-transform the *zomB* mutant with a plasmid containing the *zomB* gene, or the empty pVA838 plasmid. Instead, we introduced it into a more genetically amenable *S. pneumoniae* strain, D39, which does not contain ICE*Sp*23FST81 or the *zomB* gene. Introduction of the *zomB*-expressing plasmid to this strain did not increase the ability of the strain to lyse RBCs, or increase its production of Ply (Fig. 2c, d). However, introduction of the *zomB* plasmid significantly increased the transcription of the *ply* gene in D39, verifying the positive effect ZomB has on *ply* expression (Fig. 2e).

**Fig. 3.**
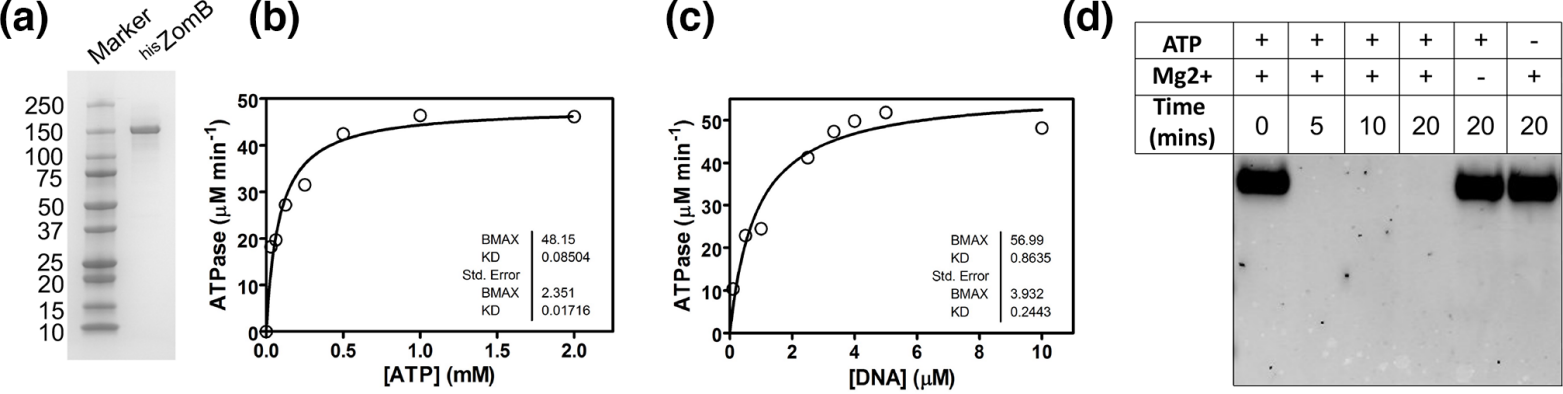
The ZomB protein has ATP-dependent nuclease activity. (**a**) An SDS-PAGE gel showing the purified His-tagged ZomB protein. (**b**) Steady-state ATPase activity of ZomB (50 nM) was measured at saturating ssDNA concentration to determine the Michaelis–Menten parameters. (**c**) Steady-state ATPase activity of ZomB was strongly stimulated by ssDNA with an apparent dissociation constant of approximately 1 µM ntds. (**d**) Nuclease assays were performed with linear DNA demonstrating that it was degraded by the ZomB protein in the presence of both ATP and divalent cations.

To characterize the biochemical activities of the ZomB protein, we expressed and purified recombinant ZomB with an N-terminal 6× histidine tag (Fig. 3a). Using a coupled assay, we found that ZomB hydrolyses ATP with Michaelis–Menten kinetics and displays a turnover number of approximately 20 s^−1^ and a *K*_m_ value of 90 µM ATP (Fig. 3b). These experiments were performed in the presence of saturating quantities of ssDNA, which was shown to strongly stimulate ATP hydrolysis with an apparent dissociation constant of approximately 1 µM (nonequilibrium thermal dissociation) (Fig. 3c). This behaviour is typical of the UvrD-like DNA helicases of which ZomB is a member [49]. To examine the putative nuclease activity, ZomB protein (50 nM) was incubated with duplex DNA with and without ATP and Mg^2+^. Degradation of the DNA by ZomB was monitored by gel electrophoresis, which showed that the protein possesses a potent ATP-dependent nuclease activity (Fig. 3d).

**Fig. 4.**
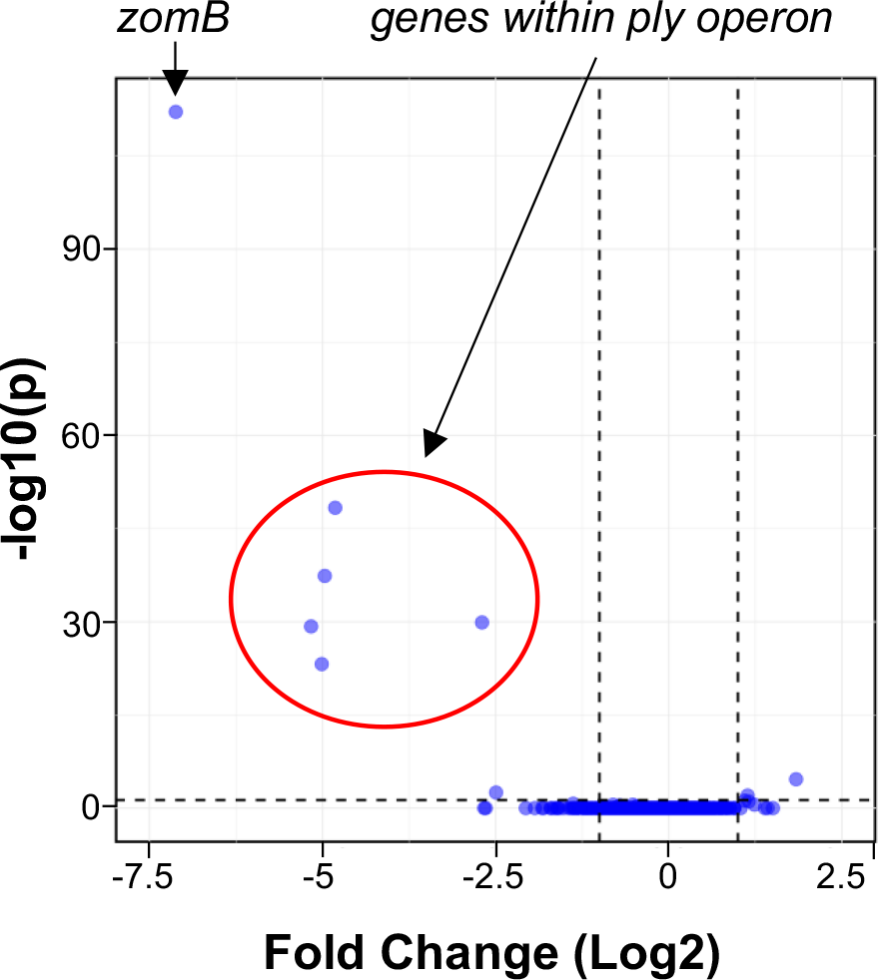
ZomB is a positive regulator of the *ply* operon. Transcription of coding regions across the wild-type *S. pneumoniae* and ZomB mutant was compared by RNA sequencing. Only nine genes were significantly affected, and of those the most affected were *zomB* and the five genes encoded on the *ply* operon.

Given the *in vitro* DNA binding and nuclease activity of ZomB, and its role in the transcription of the *ply* gene, we sought to determine the scale of its regulatory activity. To examine this, we compared the level of transcription of all *S. pneumoniae* strain ATCC 700669 coding regions between the wild-type and *zomB* mutant using RNA-sequencing. Under the growth conditions used (i.e. overnight cultures grown in THY broth), we used a >2-fold difference in expression and a *P* value of <0.05 following Benjamini–Hochberg adjustment as our significance threshold. We found that transcription of nine genes was affected by loss of the *zomB* gene, with those genes encoded within the *ply* locus being the most significantly affected (Table 3, Fig. 4).

**Table 3.** Transcriptional differences between the *zomB* mutant relative to the wild-type strain. The locus tags in bold indicate those encoded within the *ply* operon

Locus tag	Fold change (log_2_)	*P* value (adjusted)	Gene product
SPN23F11770	1.8	2.3×10^−5^	ABC-F family ATP-binding cassette domain-containing protein
SPN23F12440	1.1	9.3×10^−3^	Plasmid mobilization relaxosome protein MobC
SPN23F12470	−7.1	7.8×10^−113^	ZomB protein
**SPN23F19450**	−2.5	3.1×10^−3^	MarR family transcriptional regulator
**SPN23F19460**	−2.7	1.3×10^−30^	YebC DNA-binding transcriptional regulator
**SPN23F19470**	−4.8	4.4×10^−49^	Pneumolysin
**SPN23F19480**	−4.9	4.1×10^−38^	Hypothetical protein
**SPN23F19490**	−5.2	5.5×10^−30^	Hypothetical protein
**SPN23F19500**	−5.0	6.4×10^−24^	DUF4231 domain-containing protein

**Fig. 5.**
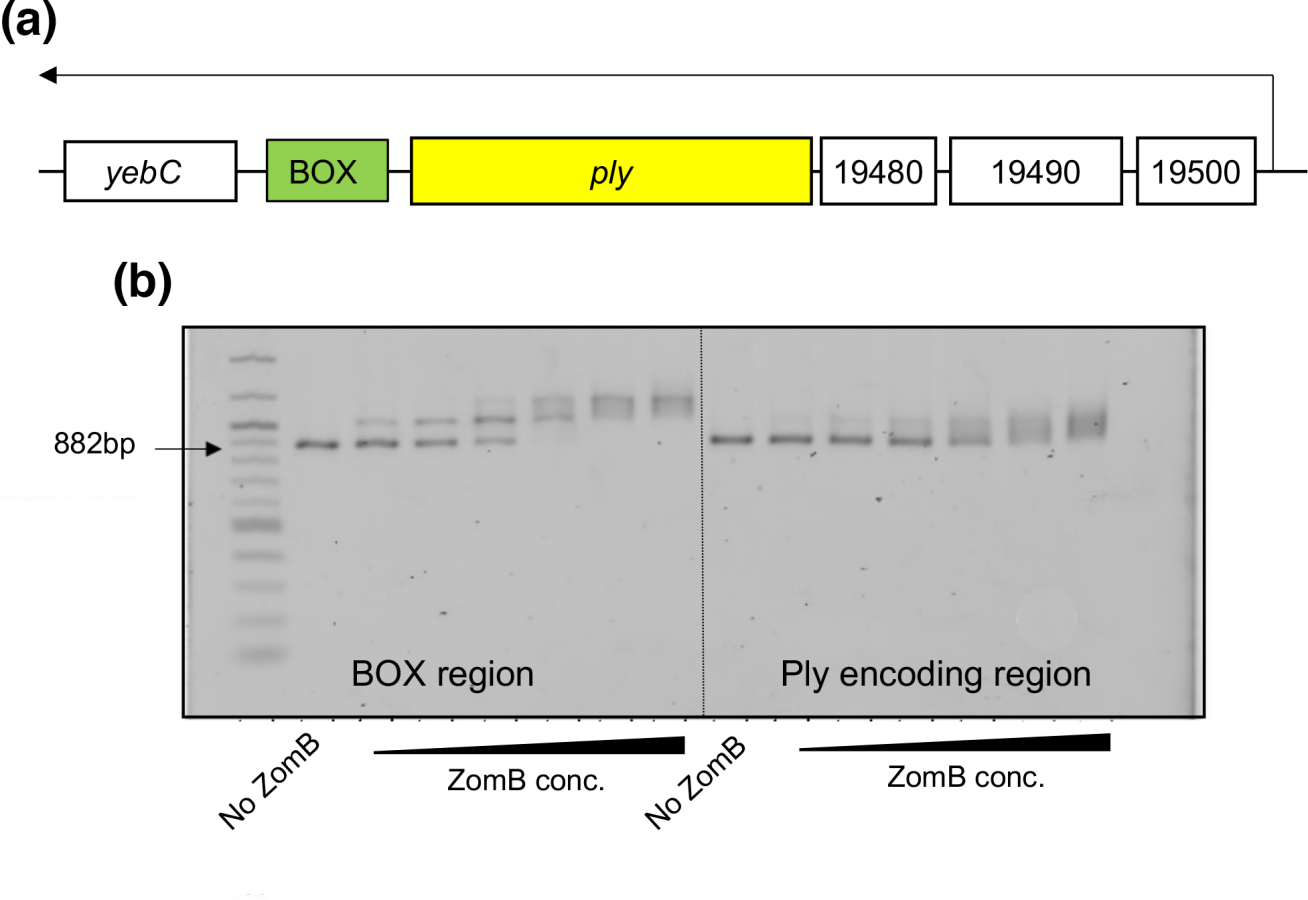
The ZomB protein specifically binds to the BOX region within the Ply-encoding operon. (**a**) Illustration of the *ply*-encoding operon with the *yebC* and *ply* genes, the SPN23F-locus tags of the neighbouring genes, and promoter and transcript length (black arrow) indicated. (**b**) EMSAs demonstrating the specificity of binding of the ZomB protein for two regions within the *ply* locus. The concentrations of ZomB used were 2.5, 5, 10, 20, 40 and 80 nM.

The *ply* gene is transcribed as part of an operon with four other genes that encode a transcriptional regulator (YebC/YeeN) and proteins implicated in the movement of Ply from the cytosol to the bacterial cell wall (SPN23F19480-19500) (Fig. 5a) [18, 19]. The operon also contains a BOX repeat region immediately downstream of the *ply* gene. Due to their internal repeating sequences, BOX regions can form stable secondary structures, and their presence has been associated with altered transcription of neighbouring genes [50, 51]. The molecular details of how their presence affects gene transcription has not yet been determined, but it is probably due to these secondary structures where they can either enhance or interfere with transcription processes depending on their relative positioning [51]. As ZomB appears to be a positive effector of *ply* transcription, and given its probable DNA binding capability, we hypothesized that it may directly interact with the *ply* locus, perhaps via its BOX region. To test this we amplified two regions of DNA from within the *ply* locus, one containing the BOX element from within the *ply* operon, as well as an equivalently sized region of DNA within the *ply* coding region, and performed electrophoretic mobility shift assays (EMSAs) with increasing concentrations of ZomB protein. As visualized in Fig. 5(b, c), the ZomB protein caused a shift in size of both regions of DNA with a higher level of affinity for the BOX-containing DNA evidenced by the shift occurring at lower concentrations of protein and with a clearer (less fuzzy) shift in the DNA. While it is tempting to speculate from this that the effect of ZomB on the transcription of the *ply* locus might be mediated via the BOX region, that the transcription of none of the genes neighbouring the other 136 BOX regions scattered across the *S. pneumoniae* genome was affected by the loss of the *zomB* gene does not support this (Table 3, Fig. 4). What is clear from this analysis, however, is that the ZomB protein can bind to at least two regions within the *ply* locus with high affinity, and this is likely to be the means by which it affects the transcription of these genes.

**Fig. 6.**
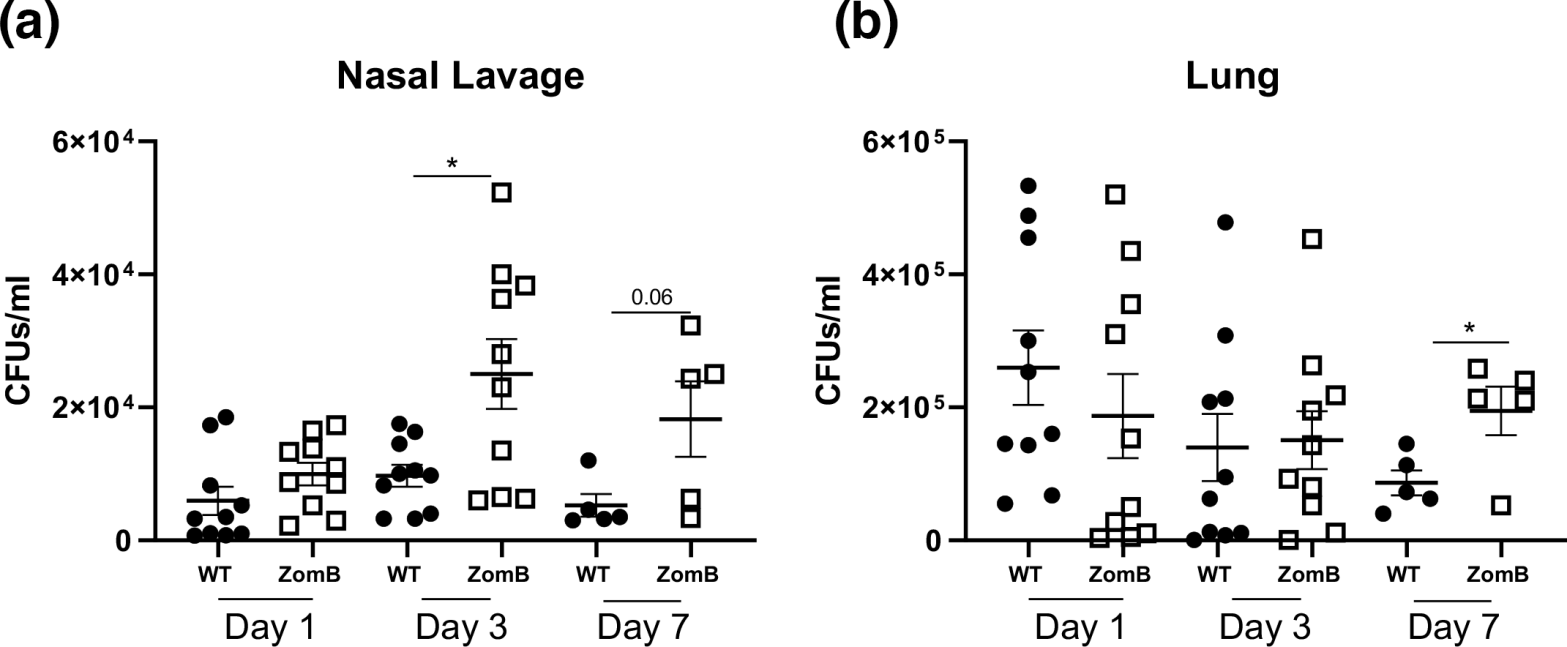
ZomB has a negative effect on the nasal colonization of mice by *S. pneumoniae *. (**a**) Groups of C57Blk mice were inoculated intranasally with wild-type *S. pneumoniae* or an isogenic ZomB mutant. At specific time points post-colonization the upper respiratory tract was lavaged with sterile PBS and the bacterial burdens in the lavage fluid were quantified. (**b**) At specific times the lungs were removed and homogenized and the bacterial burdens were quantified to determine lower respiratory tract colonization levels. The data from each mouse and sample are provided with the mean c.f.u.±sem indicated. Statistical analysis was performed using a Kruskal–Wallis test with Dunn’s multiple comparisons. *P<0.05.

The ability of pathogens to alter expression of key virulence factors to counteract host immune responses is a critical strategy of disease tolerance employed by obligate symbionts, such as *S. pneumoniae,* to facilitate persistence within its host [52, 53]. In the absence of Ply a reduced local pro-inflammatory response [13, 54] coupled with the potential for a more intracellular lifestyle [55] has been shown to promote persistence both in the nasopharynx and in the lungs. With Ply expression inversely correlated with colonization, we sought to determine how ZomB, a regulator of *ply* transcription, would affect *S. pneumoniae* colonization in a murine model. Mice were inoculated intra-nasally with a sub-lethal dose of either the wild-type or ZomB mutant and nasopharyngeal bacterial burden was monitored over 7 days (Fig. 6). The ZomB mutant demonstrated increased persistence within the nasopharynx compared to the wild-type strain with increased numbers of ZomB mutant bacteria recovered from the upper respiratory tract at days 3 and 7 post-inoculation (Fig. 6a). Consistent with this, significantly increased levels of pneumococci were also recovered from the lungs of the ZomB mutant-challenged animals compared to animals challenged with the wild-type strains on day 7 post-colonization (Fig. 6b). These finding suggest the loss of ZomB and the subsequent effect this has on Ply production affects the ability of *S. pneumoniae* to persist in a colonization state in a murine host.

## Discussion

Through the application of a functional genomics approach to a large collection of sequenced *S. pneumoniae* isolates, we have identified >100 novel putative affectors of Ply activity (Tables S2–S4). Of the associated loci, we have determined the molecular detail of the interaction between the ZomB protein encoded on ICE*Sp*23FST81 and Ply activity, where ZomB acts as a positive transcriptional regulator of the *ply* operon. While we were unable to fully complement the cytolytic activity of the ZomB mutant, by introducing the ZomB into a naive host (strain D39) we were able to verify the effect ZomB has on transcription of the *ply* gene. It is possible that the increase in *ply* transcription in strain D39 did not affect the level of Ply production in this strain due to it being an inherently high Ply producer, where its protein translational machinery or its secretory mechanism may already be working at a maximal level. We have also demonstrated that ZomB has ATP-dependent nuclease activity and that it can bind with high affinity to multiple regions within the *ply* locus. Further work is required to understand how the binding of ZomB to this locus affect its transcription, but we hypothesize that it may work in conjunction with other factors to elicit this effect.

Of the other associated loci, the most significant one identified by all three GWAS methods was the *pbpX* gene. However, we were unable to functionally validate this association through mutation and observed no association between β-lactam resistance levels and Ply activity, so as yet we do not understand the basis of this association. One hypothesis is that the polymorphisms in the *pbpX* gene affect the stem peptide composition of peptidoglycan, as this has been shown to inhibit the release of Ply from the bacterial cells [56]. Further work to test this hypothesis is currently underway. Amongst the other loci identified by all three GWAS approaches were *bgaA* and *msmG*, two genes involved in carbohydrate utilization. BgaA is a surface-expressed β-galactosidase known to play a role in pneumococcal growth, resistance to opsonophagocytic killing and adherence [57], whereas MsmG is part of the multiple sugar metabolism system [58]. A close relationship between metabolism and virulence is well established for other bacterial pathogens [59–61], where a clear link between capsule production and metabolism has been established for the pneumococci [62]. This work suggests that the effect of pneumococcal metabolism on its virulence may extend beyond capsule production and include an effect of Ply production, which is currently under investigation.

While GWAS is a powerful approach to identify novel loci associated with specific bacterial traits, there are limitations to its utility. The first is that function-altering polymorphisms in affector genes have to exist within a population for the process to identify them. For example, we were initially surprised that our approach did not identify any of the genes within the *ply* locus as associated with Ply activity, until we examined the sequence at this region and found it to be highly conserved. Having sequenced and phenotyped a collection of isolates, the next consideration is which GWAS approach to use. In our experience, work related to collecting, sequencing and phenotyping the isolates is the most labour-intensive part of the process compared to the generally quick and user-friendly GWAS pipelines that have been developed. For this reason we utilized in this study three different approaches, which allowed us to consider a wider range of loci with a focus on loci identified by more than one of the GWAS approaches. Our usual approach of functionally validating identified associations [63–67] has proven to be challenging in *S. pneumoniae *, as our clinical strains are highly resistant to transformation and the more genetically amenable laboratory strains do not always contain the associated gene, as we found in the case of *zomB*. Nevertheless, this functional genomics approach has opened up a much-neglected aspect of the biology of a major human pathogen, and provides a footing on which to base future work as we characterize the regulation of the expression of this important virulence factor.

In this work we have identified and characterized a gene encoded on an MGE with specific and targeted activity for an operon that is critical to several aspects of the biology of *S. pneumoniae *. The PMEN1 lineage is believed to have emerged from a relatively unremarkable background lineage (Clonal Complex 66) to become a globally successful pathogenic lineage, and this is at least partially attributed to the acquisition of ICE*Sp23*FST81 [8, 29, 30]. This MGE, which is present across the whole PMEN1 lineage, confers resistance to tetracycline and chloramphenicol and thus provides a clear benefit upon exposure to these antibiotics. However, for many bacterial species antibiotic resistance often incurs a fitness cost in the absence of the antibiotic, which can be offset, for example, by reducing the energetically costly production of toxins [40, 41]. Here, in stark contrast, we observe the simultaneous acquisition of increased resistance with increased toxin production. We believe this work has uncovered an intriguing form of interdependency between a host, a bacterium and an MGE, where increased Ply expression with its important contribution to bacterial transmission has potentially converted a strain from being a stable colonizer to an efficient transmitter. The long-term benefit of this ZomB-associated conversion may override the short-term increased energetic costs, with important implications for the evolution of this globally successful pneumococcal lineage.

## Supplementary Data

Supplementary material 1

Supplementary material 2

Supplementary material 3

## References

[1] Deng X, Peirano G, Schillberg E, Mazzulli T, Gray-Owen SD (2016). Whole-genome sequencing reveals the origin and rapid evolution of an emerging outbreak strain of *Streptococcus pneumoniae* 12F. Clin Infect Dis.

[2] Ioachimescu OC, Ioachimescu AG, Iannini PB (2004). Severity scoring in community-acquired pneumonia caused by *Streptococcus pneumoniae*: a 5-year experience. Int J Antimicrob Agents.

[3] O’Brien KL, Wolfson LJ, Watt JP, Henkle E, Deloria-Knoll M (2009). Burden of disease caused by *Streptococcus pneumoniae* in children younger than 5 years: global estimates. Lancet.

[4] Canvin JR, Marvin AP, Sivakumaran M, Paton JC, Boulnois GJ (1995). The role of pneumolysin and autolysin in the pathology of pneumonia and septicemia in mice infected with a type 2 pneumococcus. J Infect Dis.

[5] Hirst RA, Kadioglu A, O’callaghan C, Andrew PW (2004). The role of pneumolysin in pneumococcal pneumonia and meningitis. Clin Exp Immunol.

[6] Hirst RA, Gosai B, Rutman A, Guerin CJ, Nicotera P (2008). *Streptococcus pneumoniae* deficient in pneumolysin or autolysin has reduced virulence in meningitis. J Infect Dis.

[7] Mitchell AM, Mitchell TJ (2010). *Streptococcus pneumoniae*: virulence factors and variation. Clin Microbiol Infect.

[8] Croucher NJ, Walker D, Romero P, Lennard N, Paterson GK (2009). Role of conjugative elements in the evolution of the multidrug-resistant pandemic clone *Streptococcus pneumoniae*
^Spain23F^ ST81. J Bacteriol.

[9] Mitchell TJ, Andrew PW, Saunders FK, Smith AN, Boulnois GJ (1991). Complement activation and antibody binding by pneumolysin via a region of the toxin homologous to a human acute-phase protein. Mol Microbiol.

[10] Paton JC, Rowan-Kelly B, Ferrante A (1984). Activation of human complement by the pneumococcal toxin pneumolysin. Infect Immun.

[11] Subramanian K, Neill DR, Malak HA, Spelmink L, Khandaker S (2019). Pneumolysin binds to the mannose receptor C type 1 (MRC-1) leading to anti-inflammatory responses and enhanced pneumococcal survival. Nat Microbiol.

[12] Kadioglu A, Taylor S, Iannelli F, Pozzi G, Mitchell TJ (2002). Upper and lower respiratory tract infection by *Streptococcus pneumoniae* is affected by pneumolysin deficiency and differences in capsule type. Infect Immun.

[13] van Rossum AMC, Lysenko ES, Weiser JN (2005). Host and bacterial factors contributing to the clearance of colonization by *Streptococcus pneumoniae* in a murine model. Infect Immun.

[14] Zafar MA, Wang Y, Hamaguchi S, Weiser JN (2017). Host-to-host transmission of *Streptococcus pneumoniae* is driven by Its inflammatory toxin, pneumolysin. Cell Host Microbe.

[15] Riegler AN, Brissac T, Gonzalez-Juarbe N, Orihuela CJ (2019). Necroptotic cell death promotes adaptive immunity against colonizing pneumococci. Front Immunol.

[16] Anderson R, Feldman C (2017). Pneumolysin as a potential therapeutic target in severe pneumococcal disease. J Infect.

[17] Jenul C, Horswill AR (2019). Regulation of *Staphylococcus aureus* virulence. Microbiol Spectr.

[18] Fernebro J, Blomberg C, Morfeldt E, Wolf-Watz H, Normark S (2008). The influence of in vitro fitness defects on pneumococcal ability to colonize and to cause invasive disease. BMC Microbiol.

[19] Kimaro Mlacha SZ, Romero-Steiner S, Hotopp JCD, Kumar N, Ishmael N (2013). Phenotypic, genomic, and transcriptional characterization of *Streptococcus pneumoniae* interacting with human pharyngeal cells. BMC Genomics.

[20] Hraiech S, Roch A, Lepidi H, Atieh T, Audoly G (2013). Impaired virulence and fitness of a colistin-resistant clinical isolate of *Acinetobacter baumannii* in a rat model of pneumonia. Antimicrob Agents Chemother.

[21] Slager J, Aprianto R, Veening JW (2018). Deep genome annotation of the opportunistic human pathogen *Streptococcus pneumoniae* D39. Nucleic Acids Res.

[22] Joyce EA, Kawale A, Censini S, Kim CC, Covacci A (2004). LuxS is required for persistent pneumococcal carriage and expression of virulence and biosynthesis genes. Infect Immun.

[23] Benton KA, Paton JC, Briles DE (1997). Differences in virulence for mice among *Streptococcus pneumoniae* strains of capsular types 2, 3, 4, 5, and 6 are not attributable to differences in pneumolysin production. Infect Immun.

[24] Balachandran P, Hollingshead SK, Paton JC, Briles DE (2001). The autolytic enzyme LytA of *Streptococcus pneumoniae* is not responsible for releasing pneumolysin. J Bacteriol.

[25] Vidal JE, Ludewick HP, Kunkel RM, Zähner D, Klugman KP (2011). The LuxS-dependent quorum-sensing system regulates early biofilm formation by *Streptococcus pneumoniae* strain D39. Infect Immun.

[26] Hendriksen WT, Silva N, Bootsma HJ, Blue CE, Paterson GK (2007). Regulation of gene expression in *Streptococcus pneumoniae* by response regulator 09 is strain dependent. J Bacteriol.

[27] Walker JA, Allen RL, Falmagne P, Johnson MK, Boulnois GJ (1987). Molecular cloning, characterization, and complete nucleotide sequence of the gene for pneumolysin, the sulfhydryl-activated toxin of *Streptococcus pneumoniae*. Infect Immun.

[28] Bandara M, Skehel JM, Kadioglu A, Collinson I, Nobbs AH (2017). The accessory Sec system (SecY2A2) in *Streptococcus pneumoniae* is involved in export of pneumolysin toxin, adhesion and biofilm formation. Microbes Infect.

[29] Croucher NJ, Harris SR, Fraser C, Quail MA, Burton J (2011). Rapid pneumococcal evolution in response to clinical interventions. Science.

[30] Wyres KL, Lambertsen LM, Croucher NJ, McGee L, von Gottberg A (2012). The multidrug-resistant PMEN1 pneumococcus is a paradigm for genetic success. Genome Biol.

[31] Chen L, Ge X, Xu P (2015). Identifying essential *Streptococcus sanguinis* genes using genome-wide deletion mutation. Methods Mol Biol.

[32] Macrina FL, Tobian JA, Jones KR, Evans RP, Clewell DB (1982). A cloning vector able to replicate in *Escherichia coli* and *Streptococcus sanguis*. Gene.

[33] Lees JA, Galardini M, Bentley SD, Weiser JN, Corander J (2018). pyseer: a comprehensive tool for microbial pangenome-wide association studies. Bioinformatics.

[34] Livak KJ, Schmittgen TD (2001). Analysis of relative gene expression data using real-time quantitative PCR and the 2(-Delta Delta C(T)) Method. Methods.

[35] Langmead B, Salzberg SL (2012). Fast gapped-read alignment with Bowtie 2. Nat Methods.

[36] Heng L (2009). 1000 genome project data processing subgroup, the sequence alignment/map format and samtools. Bioinformatics.

[37] Liao Y, Smyth GK, Shi W (2014). featureCounts: an efficient general purpose program for assigning sequence reads to genomic features. Bioinformatics.

[38] Love MI, Huber W, Anders S (2014). Moderated estimation of fold change and dispersion for RNA-seq data with DESeq2. Genome Biol.

[39] Earle SG, Wu C-H, Charlesworth J, Stoesser N, Gordon NC (2016). Identifying lineage effects when controlling for population structure improves power in bacterial association studies. Nat Microbiol.

[40] Rudkin JK, Edwards AM, Bowden MG, Brown EL, Pozzi C (2012). Methicillin resistance reduces the virulence of healthcare-associated methicillin-resistant *Staphylococcus aureus* by interfering with the agr quorum sensing system. J Infect Dis.

[41] Collins J, Rudkin J, Recker M, Pozzi C, O’Gara JP (2010). Offsetting virulence and antibiotic resistance costs by MRSA. ISME J.

[42] Linares JF, López JA, Camafeita E, Albar JP, Rojo F (2005). Overexpression of the multidrug efflux pumps MexCD-OprJ and MexEF-OprN is associated with a reduction of type III secretion in *Pseudomonas aeruginosa*. J Bacteriol.

[43] Azoulay-Dupuis E, Rieux V, Muffat-Joly M, Bédos JP, Vallée E (2000). Relationship between capsular type, penicillin susceptibility, and virulence of human *Streptococcus pneumoniae* isolates in mice. Antimicrob Agents Chemother.

[44] Rieux V, Carbon C, Azoulay-Dupuis E (2001). Complex relationship between acquisition of beta-lactam resistance and loss of virulence in *Streptococcus pneumoniae*. J Infect Dis.

[45] Zhang J, Kasciukovic T, White MF (2012). The CRISPR associated protein Cas4 Is a 5’ to 3’ DNA exonuclease with an iron-sulfur cluster. PLoS One.

[46] Sampson TR, Saroj SD, Llewellyn AC, Tzeng Y-L, Weiss DS (2013). A CRISPR/Cas system mediates bacterial innate immune evasion and virulence. Nature.

[47] Heidrich N, Hagmann A, Bauriedl S, Vogel J, Schoen C (2019). The CRISPR/Cas system in *Neisseria meningitidis* affects bacterial adhesion to human nasopharyngeal epithelial cells. RNA Biol.

[48] Ma K, Cao Q, Luo S, Wang Z, Liu G (2018). cas9 enhances bacterial virulence by repressing the regR transcriptional regulator in *Streptococcus agalactiae*. Infect Immun.

[49] Gilhooly NS, Gwynn EJ, Dillingham MS (2013). Superfamily 1 helicases. Front Biosci (Schol Ed).

[50] Croucher NJ, Vernikos GS, Parkhill J, Bentley SD (2011). Identification, variation and transcription of pneumococcal repeat sequences. BMC Genomics.

[51] Knutsen E, Johnsborg O, Quentin Y, Claverys J-P, Håvarstein LS (2006). BOX elements modulate gene expression in *Streptococcus pneumoniae*: impact on the fine-tuning of competence development. J Bacteriol.

[52] McCarville JL, Ayres JS (2018). Disease tolerance: concept and mechanisms. Curr Opin Immunol.

[53] Neill DR, Coward WR, Gritzfeld JF, Richards L, Garcia-Garcia FJ (2014). Density and Duration of Pneumococcal Carriage Is Maintained by Transforming Growth Factor β1 and T Regulatory Cells. Am J Respir Crit Care Med.

[54] Wolf AI, Strauman MC, Mozdzanowska K, Williams KL, Osborne LC (2014). Pneumolysin expression by *Streptococcus pneumoniae* protects colonized mice from influenza virus-induced disease. Virology.

[55] Inomata M, Xu S, Chandra P, Meydani SN, Takemura G (2020). Macrophage LC3-associated phagocytosis is an immune defense against *Streptococcus pneumoniae* that diminishes with host aging. Proc Natl Acad Sci U S A.

[56] Greene NG, Narciso AR, Filipe SR, Camilli A (2015). Peptidoglycan branched stem peptides contribute to *Streptococcus pneumoniae* virulence by inhibiting pneumolysin release. PLoS Pathog.

[57] Singh AK, Pluvinage B, Higgins MA, Dalia AB, Woodiga SA (2014). Unravelling the multiple functions of the architecturally intricate *Streptococcus pneumoniae* β-galactosidase, BgaA. PLoS Pathog.

[58] Russell RR, Aduse-Opoku J, Sutcliffe IC, Tao L, Ferretti JJ (1992). A binding protein-dependent transport system in *Streptococcus mutans* responsible for multiple sugar metabolism. J Biol Chem.

[59] Stevens E, Laabei M, Gardner S, Somerville GA, Massey RC (2017). Cytolytic toxin production by *Staphylococcus aureus* is dependent upon the activity of the protoheme IX farnesyltransferase. Sci Rep.

[60] Bouillaut L, Dubois T, Sonenshein AL, Dupuy B (2015). Integration of metabolism and virulence in *Clostridium difficile*. Res Microbiol.

[61] Le Bouguénec C, Schouler C (2011). Sugar metabolism, an additional virulence factor in enterobacteria. Int J Med Microbiol.

[62] Paton JC, Trappetti C (2019). *Streptococcus pneumoniae* Capsular Polysaccharide. Microbiol Spectr.

[63] Laabei M, Recker M, Rudkin JK, Aldeljawi M, Gulay Z (2014). Predicting the virulence of MRSA from its genome sequence. Genome Res.

[64] Laabei M, Uhlemann A-C, Lowy FD, Austin ED, Yokoyama M (2015). Evolutionary trade-offs underlie the multi-faceted virulence of *Staphylococcus aureus*. PLoS Biol.

[65] Recker M, Laabei M, Toleman MS, Reuter S, Saunderson RB (2017). Clonal differences in *Staphylococcus aureus* bacteraemia-associated mortality. Nat Microbiol.

[66] Douglas EJA, Duggan S, Brignoli T, Massey RC (2021). The MpsB protein contributes to both the toxicity and immune evasion capacity of *Staphylococcus aureus*. Microbiology (Reading).

[67] Altwiley D, Brignoli T, Edwards A, Recker M, Lee JC (2021). A functional menadione biosynthesis pathway is required for capsule production by *Staphylococcus aureus*. Microbiology (Reading).

