# Supplementary material for: Targeted control of pneumolysin production by a mobile genetic element in Streptococcus pneumoniae
Source: Microb Genom. 2022 Apr 13;8(4):000784. doi: 10.1099/mgen.0.000784 (PMC9453066; doi:10.1099/mgen.0.000784)
Supplement: Supplementary material 1 [file mgen-8-0784-s001.pdf]

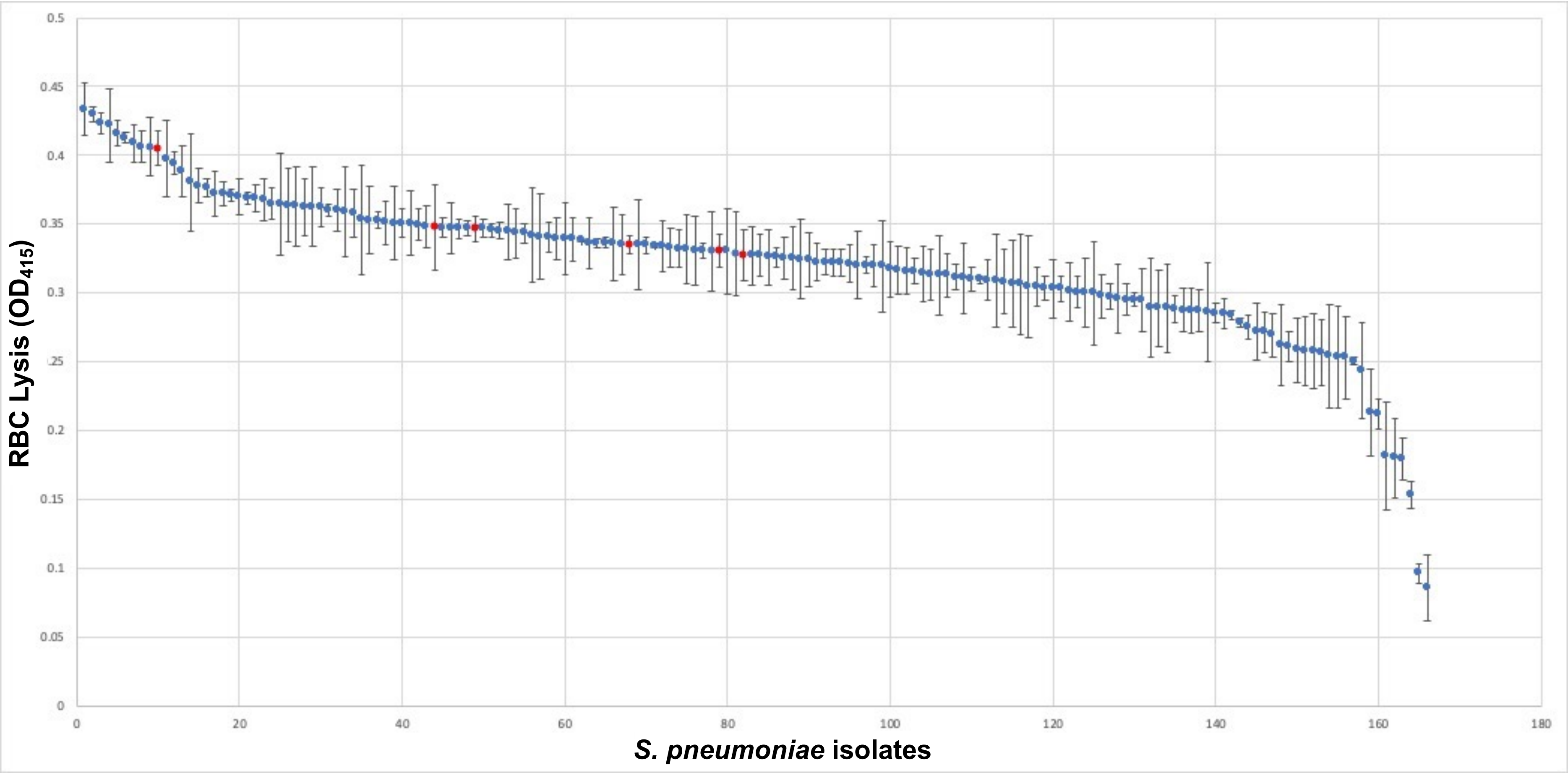

Supplementary Figure 1: Toxicity (red blood cell lysis (RBC)) of the clinical *S. pneumoniae* isolates with the isolates containing SNPs in the *zomB* gene indicated in red.
