# Supplementary material for: Targeted control of pneumolysin production by a mobile genetic element in Streptococcus pneumoniae
Source: Microb Genom. 2022 Apr 13;8(4):000784. doi: 10.1099/mgen.0.000784 (PMC9453066; doi:10.1099/mgen.0.000784)
Supplement: Supplementary material 2 [file mgen-8-0784-s002.pdf]

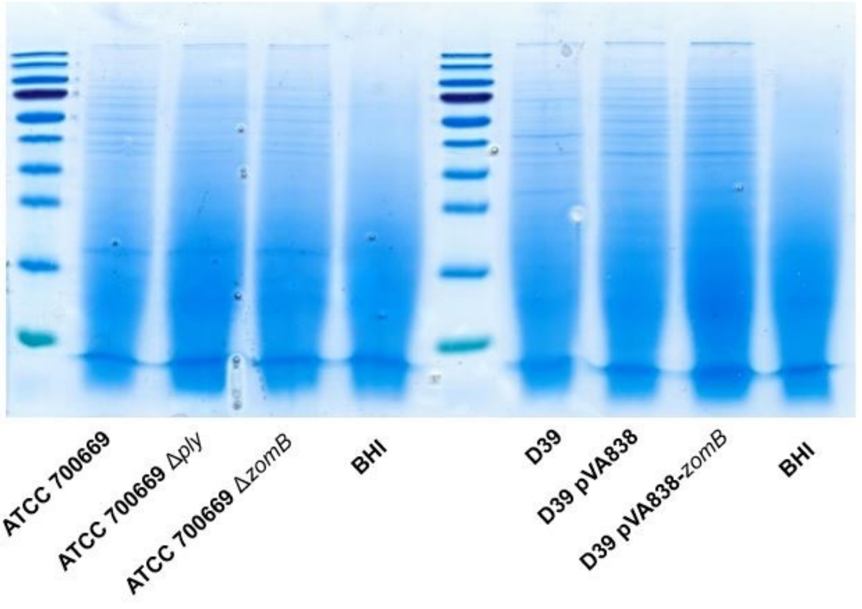

Supplementary Figure 2: Coomassie blue stained protein loading controls for the Western blots in Fig. 2d. Concentrated BHI broth is provided as a control.
